# ADP-Ribosylation Factor 6 Expression and Activation Are Reduced in Myometrium in Complicated Pregnancies

**DOI:** 10.1371/journal.pone.0037954

**Published:** 2012-05-30

**Authors:** Venkateswarlu Kanamarlapudi, Sian E. Owens, Jon Lartey, Andrés López Bernal

**Affiliations:** 1 Institute of Life Science, College of Medicine, Swansea University, Swansea, United Kingdom; 2 Division of Obstetrics and Gynaecology, School of Clinical Sciences, University of Bristol, Bristol, United Kingdom; VU University Medical Center, Netherlands

## Abstract

**Background:**

ARF6 (ADP-ribosylation factor 6) small GTP binding protein plays critical roles in actin cytoskeleton rearrangements and membrane trafficking, including internalisation of G protein coupled receptors (GPCR). ARF6 operates by cycling between GDP-bound (inactive) and GTP-bound (active) forms and is a potential regulator of GPCR-mediated uterine activity during pregnancy and labour. ARF6 contains very low intrinsic GTP binding activity and depends on GEFs (guanine nucleotide exchange factors) such as CYTH3 (cytohesin 3) to bind GTP. ARF6 and CYTH3 were originally cloned from human placenta, but there is no information on their expression in other reproductive tissues.

**Methods:**

The expression of ARF6, ARF1, and CYTH1-4 was investigated by measuring mRNA (using RT-PCR) and protein levels (using immunoblotting) in samples of myometrium obtained from non-pregnant women, and women with normal pregnancies, before or after the spontaneous onset of labour. We also analysed myometrial samples from women with spontaneous preterm labour and from women with complicated pregnancies requiring emergency preterm delivery. The GST)-effector pull down assay was used to study the presence of active ARF6 and ARF1 in all myometrial extracts.

**Results:**

ARF6, ARF1 and CYTH3 but not CYTH1, CYTH2 and CYTH4 were expressed in all samples and the levels did not change with pregnancy or labour. However, ARF6 and CYTH3 but not ARF1 levels were significantly reduced in complicated pregnancies. The alterations in the expression of ARF6 and its GEF in human myometrium indicate a potential involvement of this signalling system in modulating the response of myometrial smooth muscle in complicated pregnancies. The levels of ARF6-GTP or ARF1-GTP did not change with pregnancy or labour but ARF6-GTP levels were significantly decreased in women with severe complications of pregnancy.

**Conclusions:**

We have demonstrated a functional ARF6 system in human myometrium and a correlation between ARF6 level and activity in uterine and abnormal pregnancy.

## Introduction

Preterm birth, defined as delivery before 37 weeks of gestation, is one of the major causes of perinatal mortality and disability in surviving infants [1], [2]. Despite considerable research, the aetiology and the mechanism of preterm birth are not well understood and as a result, no satisfactory effective preventative therapy has been developed. There is a need to improve our understanding of the intracellular molecular events involved in the maintenance of uterine quiescence during pregnancy, and in initiating successful parturition at term, in order to establish the basis for the development of new therapeutic strategies to prevent preterm birth.

The myometrial smooth muscle layer of the uterus remains relatively relaxed and unresponsive during pregnancy, while it becomes highly contractile at the time of parturition. Smooth muscle contractility depends on the interaction between polymerised actin filaments and activated regulatory myosin light chains (MLCs). An increase in intracellular calcium (Ca^2+^)_i_ concentration driven by membrane depolarization through action potentials or generated by stimulatory GPCR activates calmodulin dependent MLC kinase, which in turn phosphorylates MLC to cause contraction. Contractions are reversed by a decline in [Ca^2+^]_i_ and the activity of myosin phosphatase (MP) [3] which dephosphorylates MLC to cause relaxation [4]. Uterine smooth muscle contractions are also regulated by several Ca^2+^ independent pathways: inhibition of MP by ROCK [5] resulting in Ca^2+^ sensitization, or through the activation of MP by cyclic nucleotide dependent kinases [6].

In the uterus, GPCRs coupled to Gs, e.g. β2- adrenoceptor (ADRB2), prostacyclin receptor (PTGIR), prostaglandin E1 receptor (PTGER)2 and luteinizing hormone chorionic gonadotropin receptor (LHCGR), promote relaxation by increasing intracellular cyclic AMP (cAMP) and activating protein kinase A (PKA) [7]. Premature desensitization or loss of signalling of any of these receptors may cause contractions resulting pre-term birth. On the other hand, increased expression of GPCRs coupled to Gq or Gi, e.g. oxytocin receptor (OXTR), PTGER1, PTGER3 and thromboxane A2 receptor (TBXA2) increase contractility through activation of the phospholipase C (PLC)/inositol (1,4,5)trisphosphate (IP3)/Ca^2+^ pathway by Gq, or by inhibiting cAMP production through Gi [8], [9]. Additional regulatory effects are obtained through GPCR dependent and independent activation of protein kinase C (PKC), the mitogen-activated protein kinase (MAPK) pathway and phospholipase D (PLD) [10]. Moreover, in addition to changes at the level of GPCR signalling, recent evidence suggests that the involvement of small GTP binding proteins and their GEFs may be crucial [3], [11]. Rho small GTP binding protein regulates actin-myosin interaction in uterine smooth muscles by activating RHO associated kinase (ROCK) [8], [12]. Moreover ARF6 small GTP binding protein may be involved in regulating uterine sensitivity to agonists but the presence of the ARF6 system in human myometrium has never been investigated [3] and this was the primary purpose of this manuscript.

**Table 1:** The primer sequences used for RT-PCR analysis of ARF6, CYTH3 and GAPDH mRNA expression in the human myometrium.

Gene	Accession number	Forward primer Reverse primer Amplimer location	AmplifiedPCR productsize (bp)	References
**ARF1**	M36340	5′-GCCAGTGTCCTTCCACCTGTC-3′ 5′GCCTCGTTCACACGCTCTCTG-3′ 50–385	336	[44]
**ARF6**	NM_001663	5′-ATGGGGAAGGTGCTATCCAAA-3′ 5′-GCAGTCCACTACGAAGATGAGACC-3′ 622–891	270	[45]
**CYTH1**	M85169	5′-TAGCTAATGAAATTGAAAACCTGGGAT-3′ 5′-TTCATGGCAATGAACCTCTCCACAGTG-3′ 200–722	523	[28]
**CYTH2**	X99753	5′-CAGTGAAGCCATGAGCGAGGT-3′ 5′-TTCATGGCCACAAAGCGCTCCAGG-3′ 127–650	524	[28]
**CYTH3**	NM_004227	5′-ATCGACAATCTAACTTCCGTA-3′ 5′-ATGGCGATGAACCGTTCTGCCG-3′ 258–766	509	[28]
**CYTH4**	NM_013385	5′-TGTTTGCCCAAATCGACTGCT-3′ 5′-TCATCCACCTTCTGCACCGAG-3′ 246–1060	815	[28]
**GAPDH**	NM_002046	5′-ACCACAGTCCATGCCATCAC-3′ 5′-TCCACCACCCTGTTGCTGTA-3′ 586–1037	452	[46]

The starting and end position of the mRNA sequences amplified by RT-PCR are numbered (amplimer location) according to the sequences in the NCBI data base.

**Figure 1:**
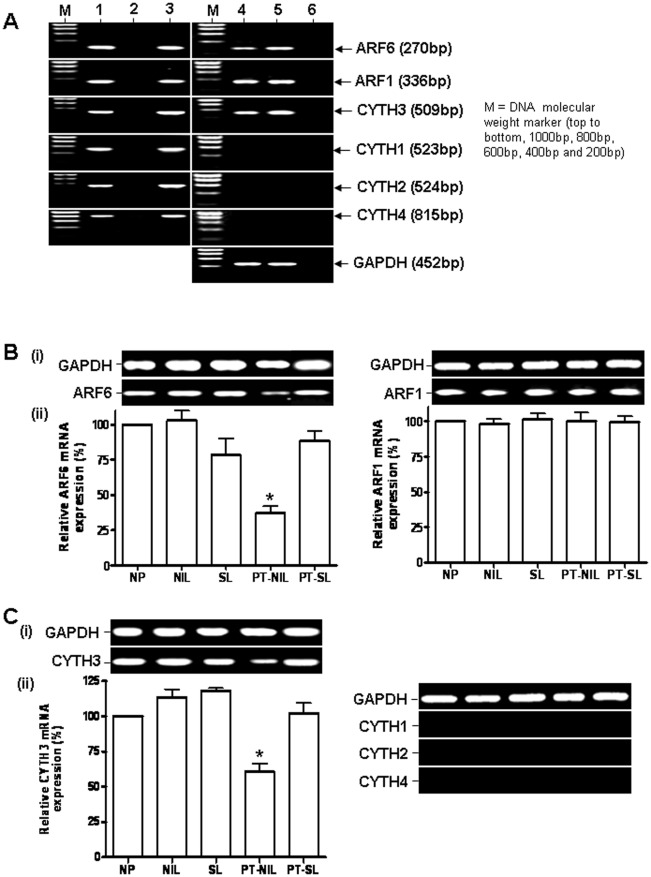
Expression of ARF1, ARF6 and CYTH3 mRNAs in human myometrium. A. RT-PCR analysis of the expression of ARF6, ARF1 and CYTH1-4 mRNAs in human placenta and myometrium. Lanes 1, MDA-MB-231 cell cDNA (+ve control), 2, water (-ve control), 3, gene specific plasmid (+ve control), 4, non-pregnant (NP) myometrium cDNA, 5, placental cDNA and 6, -RT NP myometrium cDNA (-ve control). B and C. RT-PCR analysis of ARF6/ARF1 and CYTH 1-4 mRNA expression, respectively, in human myometrium in different conditions. (i). ARF6, ARF1 and CYTH1-4 cDNA fragments were amplified from the myometrium of non-pregnant (NP), spontaneous labour (SL), not-in labour (NIL), preterm-spontaneous labour (NP-SL) and preterm- not in labour (PT-NIL) by PCR using the specific primers shown in Table 1. The intensity of the bands was quantified by densitometric scanning. The expression levels of ARF6, ARF1 and CYTH3 mRNAs were normalised to the amount of GAPDH in each sample and expressed as percentage relative to the NP expression (shown as 100%). The histogram represents means ± SEM of 4 samples for each condition (ii). *represents P<0.001, One way ANOVA with Tukey’s multiple comparison test.

ARF6 is a member of the ARF family of small GTP binding proteins, which regulate intracellular membrane traffic by cycling between active GTP- and inactive GDP-bound forms. ARFs depend on guanine nucleotide exchange factors (GEFs) for activation and GTPase activating proteins (GAPs) for inactivation. Among the six known mammalian ARF isoforms (ARFs1-6), ARF1 and ARF6 are the best characterised. ARF1 localises to and acts at the Golgi whereas ARF6 localises to and acts at the cell periphery [13]. ARF6 plays critical roles in actin cytoskeleton rearrangements and membrane trafficking, including internalization of GPCR [14]. ARF6 is activated by the cytohesin family of GEFs, which consists of four members (CYTH1-4) in humans [13]. ARF6 and CYTH3 (also known as GRP1 [general receptor for phosphoinositides 1] or PSCD3 [pleckstrin homology, Sec7 and coiled-coil domains 3]) are expressed at very high levels in human placenta from which they were originally cloned [15]. ARF6 has cellular functions that may modulate uterine activity during pregnancy and labour, for instance it inhibits LPA-induced stress fibre formation in cell models by inhibiting RHO activation [16]. In rat myometrium ARF6 is required for PLD activation [17]. ARF6 interacts with the α subunit of heterotrimeric G proteins [18] and is involved in the intracellular trafficking of many GPCRs [19] including ADRB2 [20], LHCGR [14], [21] and receptors of the vasopressin/oxytocin family [19].

The aim of this study was to assess ARF1, ARF6 and CYTH1-4 expression and ARF1 and ARF6 activation in myometrium in various conditions of pregnancy and labour. We were able to demonstrate for the first time the presence of ARF1, ARF6 and CYTH3 but not CYTH1, CYTH2 and CYTH4 mRNA and their protein products in non-pregnant and pregnant myometrial tissues. We have also measured the level of GTP-bound ARF1 and ARF6 in myometrium and found that the levels are normally high, suggesting a functional involvement of these GTPases in the uterus, however the levels of total ARF6, ARF6-GTP and CYTH3 fall significantly in association with pregnancy complications that result in emergency preterm deliveries.

## Materials and Methods

### Reagents and Antibodies

An anti-ARF6 mouse monoclonal and an anti-CYTH3 goat polyclonal antibodies were from SantaCruz Biotech. An anti-CYTH1 mouse monoclonal and an anti-ARF1 rabbit polyclonal antibodies were gift from Prof. Sylvain Bourgoin (Laval University, Quebec, Canada). An anti-CYTH2 and an anti-α tubulin mouse monoclonal antibodies were purchased from Sigma. An anti-CYTH4 rabbit polyclonal antibody was from Abcam. HRP-conjugated anti-mouse, anti-rabbit and anti-goat IgG, ECL reagents and Glutathione Sepharose beads were from GE Healthcare. All others chemicals were from Sigma unless otherwise specified.

### Cell Culture

MDA-MB-231 breast cancer cells were cultured in Dulbecco’s modified Eagle’s medium (DMEM) containing 2 mM glutamine, 100 U/ml of penicillin, 0.1 mg/ml of streptomycin sulphate and 10% fetal calf serum under 5% CO_2_ at 37°C.

### Myometrial Tissue Collection

Myometrial samples were obtained, as described previously [8], from non-pregnant (NP) pre-menopausal women who were undergoing hysterectomy for benign gynaecological disorders including fibroids or menorrhagia, and from pregnant women at term (range 37–40 weeks of gestation) who had elective caesarean sections performed before the onset of labour (not in labour; NIL), or emergency sections during spontaneous labour (SL). Indications for caesarean section included maternal request, breech presentation, previous caesarean section or placenta praevia. Labour was defined by regular uterine contractions and cervical dilatation >4 cm. Samples were also collected from women who had emergency caesarean sections preterm (range 30–35 weeks), either in spontaneous idiopathic preterm labour, with no evidence of infection for breech or fetal distress (PT-SL) or in the absence of labour for complications of pregnancy including intrauterine growth restriction, pre-eclampsia, or antepartum haemorrhage (PT-NIL). There were four women in each group. Myometrial strips were taken from the upper border of the uterine incision taking care to exclude serosal or decidual tissue. Tissues were rinsed quickly in normal saline and snap-frozen and stored in liquid nitrogen. The study was approved by the North Somerset and South Bristol Research Ethics Committee. Written informed consent was obtained from all women.

**Figure 2:**
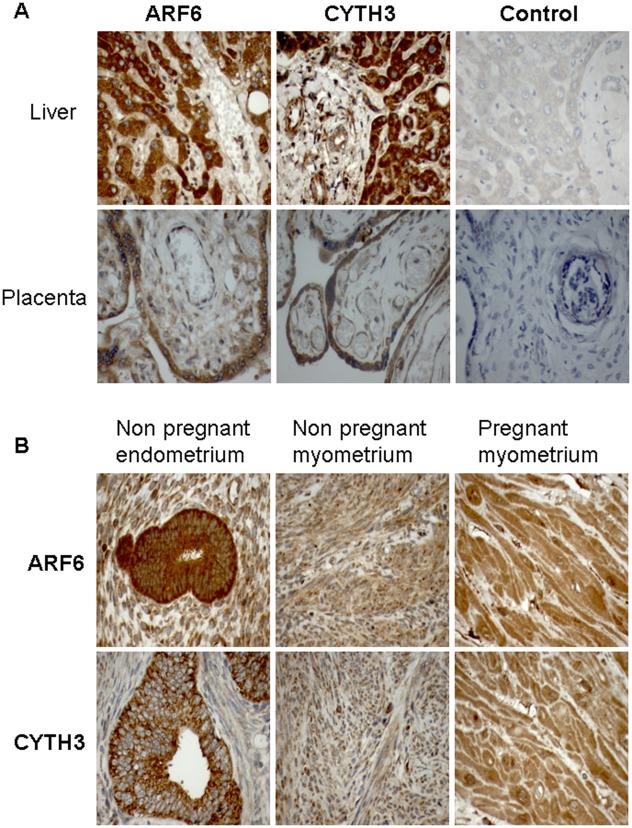
Immunohistochemical localization of ARF6 and CYTH3 in human uterine tissue sections. A. Sections of human placenta and liver show clear ARF6 and CYTH3 immunostaining. Negative controls on the right hand side were obtained with pre-immune serum. B. ARF6 and CYTH3 showing dense intracellular staining in non-pregnant endometrial glands and uniform intracellular staining pattern in both non-pregnant and pregnant myometrial cells. The images were taken at 40× magnification.

### Reverse Transcription-Polymerase Chain Reaction (RT-PCR)

Total RNA was isolated from the human myometrial tissue samples according to the manufacturer’s instructions using AurumTM total RNA Mini Kit (Biorad) and treated with DNAase I to eliminate any residual genomic DNA contamination. DNAse I treated RNA was reverse transcribed to complementary DNA (cDNA) using Superscript II Reverse Transcriptase system (Invitrogen) and by following the supplier’s instructions. Briefly, 1 µg of RNA was mixed with 50 ng of random hexamer, 10 nmoles of dNTPs (deoxyribonucleotide trisphosphates), 50 units of superscript II Reverse Transcriptase enzyme and 40 units of RNase OUT Recombinant RNase Inhibitor in a 20 µl reaction volume and incubated for 50 min at 42°C and 15 min at 70°C. The reaction mixture was then treated with 2 U of RNase H at 37°C for 20 min to degrade any un-transcribed RNA. The cDNAs samples were stored at −20°C for further use in PCR.

The ARF1, ARF6, CYTH1-4 and GAPDH gene fragments were amplified from human myometrial cDNA by PCR. The sense and antisense primers used for PCR and the PCR product sizes are shown in Table 1. PCR reaction was performed in a final volume of 25 µl containing 1 unit Taq DNA polymerase, 0.2 mM dNTPs, 0.2 µM primers and 1 µl of cDNA or 10 ng of plasmid DNA. The amplification was carried out by an initial denaturation at 94°C for 2 min followed by either 30 cycles (for plasmid DNA) or 25, 30, 35 and 40 cycles (for cDNA) of denaturation at 94°C for 30 sec, annealing at 60°C (50°C for CYTH3) for 30 sec and elongation at 72°C for 1 min. The reaction was terminated by a 5-min elongation step at 72°C. 35 cycles of the PCR reaction had the linear range of amplification of cDNA and was used for subsequent PCR reactions. The specificity of PCR was assessed by testing for genomic DNA contamination using two controls: cDNA samples prepared by RT without adding reverse transcriptase and water in place of cDNA. RT-PCR reactions were carried out only with the samples that were free of DNA contamination.

**Figure 3:**
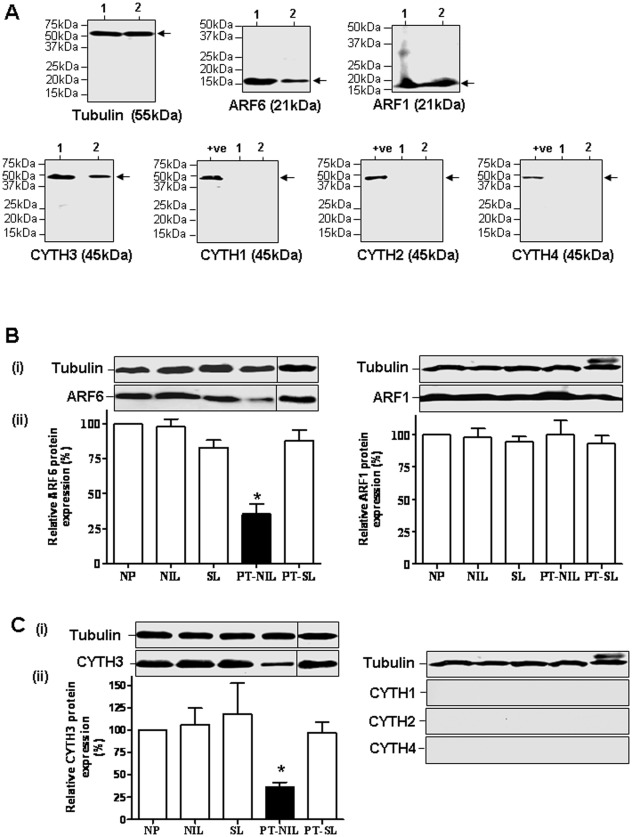
Expression of ARF1, ARF6 and CYTH3 proteins in human myometrium. A. Immunoblot analysis of the expression of ARF6, ARF1, CYTH1-4 and tubulin proteins in human placenta and myometrium. Lanes 1, Placental lysate, and 2, NP myometrium lysate. Immunoblot analysis of ARF6 and ARF6 (B) and CYTH1-4. (C) protein expression in human myometrium of NP, NIL, SL, NP-SL and PT-NIL. The tissue lysate proteins (50 µg) were separated by SDS-PAGE, blotted onto PVDF membranes, and probed with the indicated antibody (i). The lysate of MDA-MB-231 cells was used as a positive control (+ve) where indicated. The intensity of the bands was quantified by densitometric scanning. The data were normalised to the amount of tubulin in each sample and shown as means ± SE of 4 samples in the bottom graph (ii).

### Western Blotting

Myometrial tissue ground into a fine powder in liquid nitrogen was homogenised in ice-cold modified RIPA lysis buffer (50 mM Tris-HCL, pH 7.5, 150 mM NaCl, 1% Triton x-100, 0.5% (w/v) sodium deoxycholate, 0.1% (w/v) SDS, 10 mM MgCl_2_) with 1% protease inhibitors mix (Sigma). MDA-MB-231 cells were also lysed in the RIPA lysis buffer. Protein concentration of the lysates was determined using the bicinchoninic acid protein assay (Sigma) as per the manufacturer’s instructions using BSA as a protein standard. Tissue homogenate or cell lysate proteins (50 µg) were separated using SDS-PAGE and transferred to a polyvinylidene fluoride (PVDF) membrane (Millipore) in ice-cold transfer buffer (25 mM Tris, 125 mM Glycine and 20% methanol) by using semidry blotting system (Biorad). All the incubations were carried out at room temperature unless otherwise specified. The binding sites on membrane were blocked using BLOTTO (5% [w/v] skimmed milk powder in TBS-T, 0.1% Tween-20 in TBS) for one hour before incubating with a primary antibody (an anti-ARF6 [1∶200 dil.], an anti-ARF1 [1∶2000 dil.], an anti-CYTH1 [1∶500 dil.], an anti-CYTH2 [1∶500 dil.], an anti-CYTH3 [1∶1000 dil.], an anti-CYTH4 [1∶500 dil.] or mouse monoclonal anti-α-tubulin [1∶10,000 dil.]) overnight at 4°C in BLOTTO. The blots were then washed in TBS-T for 15 minutes for three times and incubated with HRP-conjugated an anti-mouse or anti-rabbit or anti-goat IgG antibody (1∶2500 dil.) in BLOTTO for 1 hr. Following the wash of the membrane for 15 min for three times, the protein bands on the membrane were visualized using ECL reagent [22].

**Figure 4:**
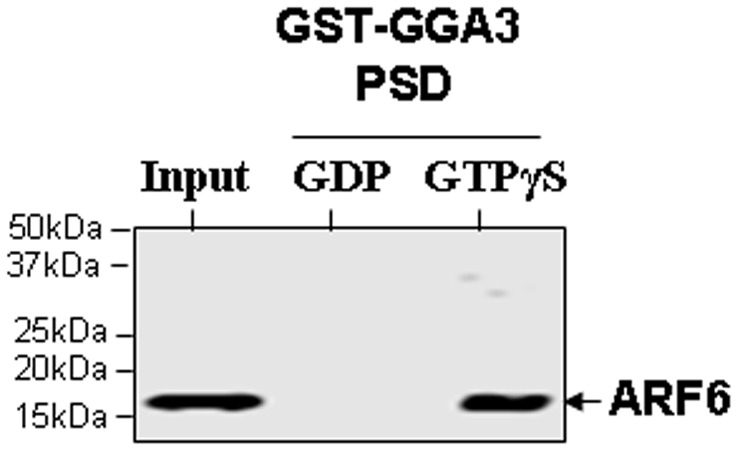
GST-GGA3 PBD binding to ARF6-GTP. Myometrium tissue isolated from non-pregnant woman was lysed and the lysate was incubated with 0.1 mM GTPγS or 1 mM GDP for 30 min. The glutathione resin coupled to GST-GGA3 PBD was added to the lysate and continued the incubation for further 2 h. After washing the beads, the protein bound to the beads were analysed by immunoblotting using an anti-ARF6 mouse monoclonal antibody. The lysates that were not incubated with beads were used as input controls in immunoblotting.

### Immunohistochemistry

Immunohistochemistry was carried out as described [8]. Briefly, paraffin-embedded tissue sections were incubated with 0.3% hydrogen peroxide for 10 min to block endogenous peroxidase activity and blocked with 2.5% horse serum for 30 min to reduce non-specific binding. The sections were incubated with primary antibody (an anti-ARF6 mouse monoclonal antibody [1∶50 dil.] or anti-CYTH3 goat polyclonal antibody [1∶200 dil.]) for 45 min at room temperature, biotinylated an anti-mouse or an anti-goat secondary antibody (Vectastain Quick Universal Kit) for 20 min, streptavidin peroxidase reagent for 20 min, and diaminobenzidine chromogen solution for 5 min, and then were counterstained in Mayers Haematoxylin for 1 min, dehydrated in alcohol, immersed in xylene, and mounted.

**Figure 5:**
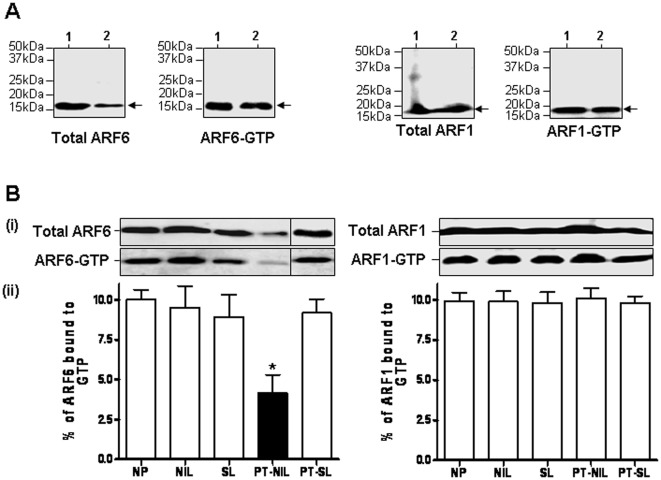
The GST-pulldown analysis of active ARF1 and ARF6 levels in the myometrium. A. Analysis of the ARF1-GTP and ARF6-GTP levels in human placenta and myometrium. Lanes 1, Placenta and 2, NP myometrium. B. The GST-pulldown analysis of active ARF1 and ARF6 levels in human myometrium of NP, NIL, SL, NP-SL and PT-NIL. The tissue lysates from NP, NIL, SL, PT-NIL and PT-SL were incubated with GST-GGA3 PBD resin to precipitate ARF1-GTP or ARF6-GTP as described in the materials and method section. The total ARF1 and ARF6 expression in the cell lysates (upper panel) and the activated ARF1 and ARF6 that was pulled down with GST-GGA3 PBD (lower panel) were detected by immunoblotting using an anti-ARF1 rabbit polyclonal and an anti-ARF6 mouse monoclonal antibody respectively (i). The intensity of the bands was quantified by densitometric scanning. The data were expressed as percentage of total ARF1 or ARF6 that is GTP bound. The histogram represents an average of 4 samples for each condition (ii).

### GST-GGA3 PBD Pull Down Assay

The glutathione S-transferase (GST)-Golgi associated, gamma adaptin ear containing, ARF binding protein 3 (GGA3) protein binding domain (PBD; amino acids 1–316) pull-down assay was performed as described [23], [24]. Since the GGA3 PBD pull-downs the active form of all ARF isoforms, only Western blotting of the pull downs with an anti-ARF6 or an anti-ARF1 antibody makes the assay specific for ARF1-GTP or ARF6-GTP. The myometrial tissue lysates, prepared as described above, equivalent to 0.3 mg of protein, were incubated for 2 hr at 4°C with 15 µl of glutathione resin coupled to GST-GGA3 PBD fusion protein (3 mg/ml of resin). The resin was then washed three times with wash buffer (50 mM Tris-HCl, pH 7.5, 10 mM MgCl_2_, 150 mM NaCl and 1% Triton X-100). The tissue lysates that were not incubated with the resin were used as an input controls. The Input and pull-down resin samples were boiled in the SDS-PAGE sample buffer (62.5 mM Tris-HCL pH 6.8, 10% Glycerol, 2% SDS, 5% 2-mercaptoethanol and 0.01% Bromophenol Blue) and analysed by immunoblotting using an anti-ARF1 or an anti-ARF6 antibody. Immunoblots were scanned to quantify the intensity of ARF bands and to calculate the percentage of ARF1 or ARF6 precipitated from the lysates of myometrial tissues with GST-GGA3 PBD resin. The GST-GGA3 PBD fusion protein coupled to glutathione beads as described elsewhere [25].

### Densitometry Analysis

Image-J programme was used for densitometric analysis. Messenger RNA and protein expression levels in different myometrial samples were normalised using house-keeping gene GAPDH mRNA and house-keeping protein α-tubulin, respectively. The expression levels were plotted in comparison to the NP value (100%) where indicated and represented graphically.

### Statistical Analysis

The data were analysed using the Graph Pad Prism software. Multiple group comparison were performed by using the analysis of variance (ANOVA) and compared with Tukey post-hoc comparison. P<0.05 was considered to be statistically significant.

## Results

### Expression of ARF1, ARF6 and CYTH3 mRNAs in Human Myometrium and Placental Tissue

We investigated first the expression of ARF1, ARF6, CYTH1, CYTH2, CYTH3 and CYTH4 mRNAs in myometrium by RT-PCR (Figure 1). Human placental cDNA, where the expression of ARF6 and CYTH3 mRNAs has been previously reported, MDA-MB-231 breast cancer cells (which known to express ARF1, ARF6 and CYTH1-4) cDNA and plasmid carrying ARF1, ARF6, CYTH1, CYTH2, CYTH3 or CYTH4 cDNA were used as positive controls along with water and –RT cDNA as negative controls in the RT-PCR analysis [23], [26], [27], [28]. RT-PCR products of the expected size were obtained only for ARF1, ARF6 and CYTH3 in myometrium, demonstrating the expression of ARF1, ARF6 and CYTH3 mRNAs in human myometrium.

### ARF1, ARF6 and CYTH3 Gene Expression in Myometrium does Not Change with Pregnancy or Labour but the Expression of ARF6 and CYTH3 is Decreased in Complicated Pregnancies

RNA isolated from myometrial tissue collected from the five groups of women, NP, NIL, SL, PT-NIL and PT-SL, was subjected to RT-PCR analysis to determine whether there are any alterations in ARF1, ARF6, CYTH1-4 genes expression during pregnancy or labour. Transcripts (mRNAs) for ARF1, ARF6 and CYTH3 were seen in all the myometrial tissues studied. The expression of ARF1, ARF6 and CYTH3 mRNAs in the NIL and SL was comparable with that in NP, suggesting that neither pregnancy nor the onset of labour have any effect on the regulation of these genes. However a significant decrease was observed in the expression of transcripts for ARF6 and CYTH3 in the PT-NIL group in comparison to the other groups (Figure 1B, C), indicating a possible association between severe complications of pregnancy and alterations in ARF6 function.

### Immuno-histochemical Analysis of ARF6 and CYTH3 in Uterine Tissue Sections

The presence of ARF6 and CYTH3 expression was confirmed by immunohistochemistry. Figure 2 (top panel) shows third trimester placental villi and human liver stained as positive controls. In NP uterine sections, there was intense staining of ARF6 and CYTH3 in the endometrial glands (Figure 2, bottom panel). Moreover, positive staining was seen for ARF6 and CYTH3 in both non-pregnant and pregnant myometrial cells, confirming the validity of the RT-PCR and immunoblotting data.

### ARF1, ARF6 and CYTH3 Protein Expression in Myometrium does Not Change with Pregnancy or Labour but the Expression of ARF6 and CYTH3 Proteins is Decreased in Complicated Pregnancies

ARF1, ARF6, CYTH1-4 proteins expression in myometrium and placenta was analysed by immunoblotting (Figure 3A). The bands were obtained only for ARF1, ARF6 and CYTH3 in positive control placenta and myometrium, indicating the expression of ARF1, ARF6 and CYTH3 but not CYTH1, CYTH2 and CYTH4 proteins in human myometrium. ARF6 and CYTH3 proteins were expressed in myometrium at lower levels than in placenta. Although ARF1 and ARF6 are 21 kDa proteins, their bands appear at a lower position in an immunoblot than expected due to the N-terminal myristoylation.

Immunoblotting analysis of the five groups of women, NP, NIL, SL, PT-NIL and PT-SL demonstrated ARF1, ARF6 and CYTH3 but not CYTH1, CYTH1 and CYTH4 protein expression in all the groups studied (Figure 3B, C). In agreement with the mRNA results, myometrial ARF1, ARF6 and CYTH3 protein expression did not change with pregnancy or with the onset of term or preterm labour. Moreover a significant decrease in both ARF6 and CYTH3 expression was noticed in the PT-NIL group in comparison to the other groups, confirming that severe complications of pregnancy may affect myometrial ARF6 expression.

### ARF6-GTP Levels in Pregnant and Non-pregnant Myometrium

The active form of ARF6, ARF6-GTP, interacts with downstream effectors to promote reorganisation of actin cytoskeleton. In order to confirm the functional relevance of the mRNA and protein expression data, we measured the active GTP-bound fraction of ARF6 and ARF1 in myometrial extracts. To establish the validity of the PBD of the human GGA3 effector protein as a probe to isolate specifically the active GTP-bound form of ARF6, we pre-treated human myometrial tissue lysates with either GTPγS or GDP to activate or inactivate ARF6, respectively, and incubated with GST-GGA3 PBD coupled to glutathione beads. The protein bound to the beads was analysed by immunoblotting using ARF6 antibody. As shown in Figure 4. ARF6 in GTP-bound form, but not in the GDP-bound form, was specifically pulled-down by the GST-GGA3 PBD beads.

We were able to precipitate ARF6-GTP and ARF1-GTP from the lysates of placenta and the myometrial lysates in all the groups of women (Figure 5), confirming the presence of active ARF1 and ARF6 in non-pregnant and pregnant myometrial tissue and placenta. ARF1-GTP and ARF6-GTP levels (approximately 10% of total ARF) remained relatively constant in pregnancy and labour, but ARF6-GTP levels were significantly decreased in the PT-NIL group (Figure 5).

## Discussion

Our study demonstrates for first time the expression of ARF1, ARF6 and CYTH3 but not the other CYTH family members in human myometrium. Moreover we were able to demonstrate with a GST-effector pull down assay that the GTP-bound ARF6 and ARF1 are present in the tissue, confirming that an active form of ARF1 and ARF6 are likely to be involved in myometrial function. The levels of ARF1, ARF6 and CYTH3 remain stable during pregnancy and labour and the potential role of these proteins in the maintenance of gestation and the onset of parturition is only speculative at this stage. RHO proteins have a role in actin dynamics. Lysophosphatidic acid (LPA)-stimulated stress fibre formation in Swiss 3T3 cells is mediated by RHO [29] and involves activation of the PLD signalling pathway [30]. PLD hydrolyses phosphatidyl choline (PC), the major phospholipids of membranes, to phosphatidic acid (PA) and choline. PA is involved in actin reorganization [31]. Recently the role of ARF6 in the PLD signalling pathway has been implicated as the active ARF6 mutant (ARF6 Q67L) co-localizes with PLD at the plasma membrane in Hela cells [32]. CHO cells over-expressing the active ARF6 mutant do not exhibit stress fibre formation in response to LPA in comparison to non-transfected cells but there was a dramatic effect on the expression level of RHOA. In this system activation of ARF6 down regulates RHO signalling [16]. In rat myometrium, ARF6 was detected exclusively in the membrane fraction and regulates PLD activity through a mechanism which involves G protein βγ subunits. [17]. The involvement of ARF6 in PLD activity is of relevance to myometrial responses to endothelin and tyrosine kinase receptors in rat [17] but such involvement has not been investigated in human myometrium. From the information available a direct effect of the ARF6 system in the biochemical events that trigger term or preterm labour cannot be proposed, however these are biologically active molecules and further research is necessary to understand the reason for their presence in myometrial tissue and alterations in their expression in complicated pregnancies.

We have demonstrated an increase in active GTP-bound RHOA in spontaneous preterm labour myometrium [8]. The active RHOA enhances myometrium contractility by inhibiting myosin phosphatase through ROCK, and this pathway may be involved as one of the causative factors in preterm labour. Since ARF6 activation antagonises RHO-mediated activities, it is possible that changes in the expression of the ARF6/CYTH3, and/or ARF6 activation may play a role the development of premature uterine contractions by increasing RHO activation in human myometrium. Our data show that ARF6 and CYTH3 expression levels remain relatively constant during normal pregnancy and labour and we have no evidence of changes in ARF6-GTP that might relate to alterations in RHO signalling. Further work is required to investigate the relationship between ARF6 and RHO in human myometrium.

The regulation of uterine contractility involves input from many GPCRs present in myometrial cells. In general, stimulatory receptors promote contractility by increasing intracellular calcium through a Gq/PLC/PKC pathway whereas inhibitory receptors promote relaxation by activating the Gs/adenylyl cyclase/cAMP/PKA pathway. Additional regulatory input is provided by pertussis toxin sensitive G proteins of the Gi/o family which are substrates for ADP-ribosylating agents. Gi/o proteins inhibit adenylyl cyclase and potentiate the stimulatory effect of alpha2A adrenoceptor (ADRA2A) [33] and OXTR [34]. Uterine GPCRs include receptors for endogenous stimulatory agonists such as oxytocin and endothelin as well as inhibitory receptors such as PTGER2, ADRB2 and LHCGR [8]. ARF6 may have a dual role in pregnancy: firstly, by promoting uterine quiescence during ongoing gestation by favouring the desensitization and internalization of stimulatory receptors [35] and secondly by decreasing the availability of inhibitory receptors [20], [36], [37] at the time of the onset of labour, when increased contractility is required. Such roles would not be necessarily reflected in changes in ARF6 mRNA or protein levels in myometrial tissue with pregnancy or labour. The role of ARF6 in GPCR desensitisation has been studied best in relation to the LHCGR in porcine ovarian cells and it involves liberation of arrestin 2 protein that inhibits the ability of the receptor to stimulate the Gs/adenylyl cyclase pathway [14]. We have recently analysed the role of ARF6 activation in human LHCGR internalisation [38], but the involvement of ARF6 in the functional regulation of stimulatory myometrial receptors other than endothelin receptor is not known and will be the subject of our future investigations.

An unexpected finding from this paper was the decrease in expression of myometrial ARF6/CYTH3 and ARF6-GTP levels in pregnancies complicated by pre-eclampsia, intrauterine growth restriction or haemorrhage. ARF6 is a ubiquitous signalling molecule and it is likely to play an important regulatory role on cell surface receptors functions in the maternal cardiovascular system, placenta and other organs involved in fetal growth and homeostasis. Moreover ARF6 can interact with other proteins, for example ARL4D which is expressed in the uterus, kidney and T lymphocytes [39]. ARF-like protein (ARL)4D can potentiate ARF6 effects through CYTH3 translocation to the cell membrane and coordinated GTPase cascades [40]. It has been shown that in pre-eclampsia there is a systemic inflammatory response with increased T cell activation [41] and in this regard ARL4D has been identified as a target for cytotoxic T lymphocytes [42]. It is possible that the decrease in myometrial ARF6 expression and activity, which we have uncovered here, is a reflection of altered ARF6 signalling in other organs. In most obstetric populations, including women attending our hospital, there is a clear link between pre-eclampsia, intrauterine growth restriction and other risk factors for preterm delivery [43] and alterations in the ARF6 pathway may be a reflection of underlying pathology, however in this study we did not have enough PT-NIL samples to carry out multivariable analysis to assess the contribution of each independent risk factor. This is an interesting challenge for future research and further studies in a large cohort of women with complicated pregnancies are warranted.

In conclusion, we have demonstrated a functional ARF6 system in human myometrium and an association between ARF6 expression and activity in myometrium and abnormal pregnancy. Moreover, the present study should pave the way to assess whether reduction in ARF6 expression and activity is the cause or consequence in complicated pregnancy. This aspect could be addressed in future using tissue specific knock out of ARF6 in mice and/or the specific chemical inhibitor, SecinH3, for the cytohesin family of ARF GEFs in pregnant mice [38].
